# Effect of Carboxymethyl chitosan-sodium alginate hydrogel loaded with *Astragalus membranaceus*-*Panax notoginseng* on wound healing in rats

**DOI:** 10.3389/fphar.2025.1526828

**Published:** 2025-01-29

**Authors:** Jing Li, Linai Li, Yue Yu, Ruixin Qin, Chaoyan Yu, Chen Chen, Youming Dong, Yadong Tan, Yunen Liu, Xuanli Liu

**Affiliations:** ^1^ Key Laboratory of Ministry of Education for TCM Viscera-State Theory and Applications, Liaoning University of Traditional Chinese Medicine, Shenyang, China; ^2^ Teaching and Research Section of The Internal Medicine of Traditional Chinese Medicine, The Affiliated Hospital of Liaoning University of Traditional Chinese Medicine, Shenyang, China; ^3^ Shuren International College, Shenyang Medical College, Shenyang, China; ^4^ Material & Process Analysis (Technologie Werkstoff - und Verfahrensanalytik), BMW Brilliance Automotive Ltd., Shenyang, China

**Keywords:** Carboxymethyl chitosan-sodium alginate hydrogel, astragali membranaceus, Panax notoginseng, rats, wound healing

## Abstract

Skin wound healing is a complex physiological process that involves many different cell types and signaling molecules. In traditional Chinese medicine, *A. membranaceus* and *Panax notoginseng* are commonly used together for the treatment of wound injury for their significant efficacy. The application of new materials may make A. *membranaceus*-P. notoginseng (AP) play a better curative effect. In this study, we fabricated a Carboxymethyl chitosan-Sodium alginate hydrogel loaded with the extract of *Astragalus membranaceus*-*P. notoginseng* (APCS), which showed favorable stability, biocompatibility, and the ability to rapidly release drugs. Cell culture experiments demonstrated that the APCS hydrogel dramatically increased fibroblast proliferation, migration, and differentiation into myofibroblast. *In vivo* experiments of wound healing on SD rats showed that APCS hydrogels significantly accelerated skin wound healing, reduced inflammatory response, enhanced the formation of blood vessels, granulation tissues, and collagen fibers, and promoted re-epithelialization at the wound site. Increased expression of catalase, VEGF, and PGP9.5 of wound tissue indicated that APCS hydrogels inhibited oxidative stress and promoted vascular and neuronal regeneration. In summary, the APCS hydrogel displayed great potential as a dressing for achieving satisfactory healing of full-thickness wounds.

## 1 Introduction

Wounds caused by physical and chemical injuries or microbial infections are inevitable events in life (Gumel et al., 2015; Zhu et al., 2023). Wound healing involves a series of overlapping phases, including hemostasis, inflammation, proliferation, and remodeling (Peña and Martin, 2024). Full-thickness skin defects have been treated with a variety of therapies, but the state of wound care today still reduces the quality of life of patients and places a heavy burden on society (Liu W. et al., 2023; Zhang et al., 2023). Therefore, effective ways of promoting skin wound healing have become a major concern in global healthcare.

Numerous medications derived from plant sources show significant effects in wound healing, and more than 80% of people worldwide use herbal remedies to treat various skin diseases (Gumel et al., 2015; Zhu et al., 2024). Specifically traditional Chinese medicine has shown wide experience in regulating the wound healing process and has displayed outstanding advantages in wound repair (Zhou et al., 2024; Chi et al., 2021; Yao et al., 2023). In traditional Chinese medicine, *A. membranaceus* Fisch. and *P. notoginseng* Burkill. are frequently used to promote skin wound healing. *A. membranaceus* has been regarded as a beneficial medication in relieving skin damage for its potent wound-healing actions (Jia et al., 2024). *P. notoginseng* has been utilized to promote blood circulation and relieve pain. The pulverous of *P. notoginseng* was frequently used to treat various bleeding wounds in ancient China (Gao et al., 2023). In the research of modern pharmacology, Astragalus polysaccharide, one of the main active ingredients of *A. membranaceus*, has shown antioxidant stress effects in wound healing (Yang et al., 2015). Astragaloside IV has been used in wound therapy for its strong anti-inflammatory and immunoregulatory properties (Luo et al., 2016). Panax notoginseng saponins obtained from *P. notoginseng* have displayed antioxidant and anti-apoptosis properties in the wound microenvironment (Lei et al., 2022).

Sustained drug release plays an important role in the wound healing process (Kaur et al., 2024). The outstanding carrier can help drugs sustainably release in wound healing and play a longer efficacy (Li et al., 2022). Carboxymethyl chitosan (CMCS) and Sodium alginate (SA) have been widely used in the field of biomaterials (bone tissue repair, chronic diabetic wounds, burn wounds, and other refractory wounds, etc.), demonstrated good biocompatibility, injectability, a wide range of origins, and other excellent properties (Liu T. et al., 2023; Xie et al., 2022; Gong et al., 2022). In our previous study, the CMCS/alginate hydrogel was a potential carrier to treat wound healing, which showed excellent water retention properties, adhesiveness, and injectability (Lv et al., 2019). In this study, we employed *A*. *membranaceus*-*P*. *notoginseng* (AP) and Carboxymethyl chitosan-Sodium alginate hydrogel (CS) to develop a composite hydrogel, investigated proliferation, migration, and differentiation of APCS on L929 cells *in vitro*, and assessed the wound healing *in vivo* by measuring neurogenesis, angiogenesis, collagen synthesis, and re-epithelialization in full-thickness wounds.

## 2 Materials and methods

### 2.1 Chemicals and reagents

Carboxymethyl chitosan (Macklin, China), Sodium alginate (Macklin, China), D-glucono-δ-lactone (GDL, Sigma Aldrich, United States), RIPA buffer (Beyotime, China). Cell Counting Kit-8 (Solarbio, China), Masson’s Trichrome Stain Kit (Solarbio, China). BCA Protein Assay Kit (Beyotime, China), Minimum essential medium (MEM, Gibco, United States), Fetal bovine serum (FBS, Gibco, United States). Clarity Western ECL Substrate (Shanghai, Bole Life Medical Products Co. Ltd., China), anti-CD31 (Abcam, UK) and α-SMA (Cell Signaling Technology, United States), VEGF (Proteintech, United States), PGP9.5 (Abcam, UK), Catalase (Cell Signaling Technology, United States), GAPDH (Proteintech, United States), anti-rabbit and anti-mouse secondary antibodies (Cell Signaling Technology, United States). All other chemicals were of analytical grade.

### 2.2 Preparation of AP, CS hydrogels, and APCS hydrogels

50 g of *A. membranaceus* and 50 g of *P. notoginseng* were extracted with 600 mL of 75% ethanol three times. The filtrates of traditional Chinese medicine were concentrated using a rotary evaporator. The concentrated extract was evaporated by a nitrogen blower until complete drying.

2 mL of 2% SA and 2 mL of 2% CS were mixed until obtaining a homogeneous suspension. 1 mL of GDL solution (20 mg/mL) was added to the CMCS/SA solution with constant stirring in the ice water bath for 1 min to form CS hydrogels.

2% SA and 2% CS were mixed separately by a filtered solution of AP (125 mg/mL). 2 mL of 2% SA-125 mg/mL AP and 2 mL of 2% CS-125 mg/mL AP were mixed until obtaining a homogeneous suspension. 1 mL GDL solution (20 mg/mL) was added to the CMCS/SA solution and maintained under stirring in the ice water bath for 1 min to form APCS hydrogels with AP concentration of 100 mg/mL.

### 2.3 Characterization of APCS hydrogels

Both the CS hydrogel and the APCS hydrogel were cut into cross-sectional slices of 2 mm thickness and 25 mm diameter. The storage modulus (G′) and loss modulus (G″) of hydrogels with a rheometer (AR 2000, TA Instruments) under the frequency range of 0.1–10 Hz were measured at 37°C and a constant strain, and a strain sweep on hydrogels were carried out from 0.1% to 10% at a frequency of 1 Hz.

The samples were tested for their compressive properties using a universal tensile testing machine (Instron3345). Hydrogel samples of diameter 5 mm and height 8 mm were placed between compression plates with 100 N load cells. The compressive stress-strain behavior of the samples was plotted and used to calculate compressive strength and modulus.

All samples were covered with a gold layer, and the structural morphology of CS hydrogels and APCS hydrogels were observed by scanning electron microscopy (SEM, inspect F50, FEI).

### 2.4 *In vitro* release study

Flux dialysis was used to conduct the drug release experiment. The dialysis bag with 2 mL of APCS was put into 50 mL of PBS at 37°C and stirred continuously with a magnetic stirrer. 1 mL was collected and immediately replaced with an equal amount of fresh PBS at 0 h, 6 h, 12 h, 18 h, 24 h, 30 h, 36 h, 42 h, and 48 h, respectively. The oleanolic acid release was obtained by detecting absorbance at 523 nm using an ultraviolet spectrophotometer (Agilent Technologies). All the experiments were repeated three times, and the cumulative drug release amount was determined with the standard calibration curve.

### 2.5 *In vitro* cytocompatibility

L929 cells were purchased from Stem Cell Bank, Chinese Academy of Sciences, and cultured in MEM with 10% FBS, 1% penicillin, and 1% streptomycin. Cells were grown at 37°C with 5% CO2. L929 cells (1 × 10^5^ cells/well) were seeded on the 96-well plate, and cultured for 24 h. The supernatant was replaced by the various concentrations (0.0125, 0.025, 0.05, 0.075, 0.15, and 0.3% v/v) of the CS/APCS hydrogel leaching solution, or the various concentrations (12.5, 25, 50, 75, 150, and 300 mg/L) of AP solution. After treatment for 48 h, 100 μL of MEM containing 10% CCK-8 reagent was added to each well. The absorbance of optical density (OD) was recorded at 450 nm.

### 2.6 Cell proliferation assay

The L929 cells were planted on the 96-well plate with a number of 1 × 10^3^ cells/well, and cultured for 24 h. The supernatant was replaced by the CS/APCS hydrogel leaching solution (0.075% v/v) or the AP solution (75 mg/L), and the cells were cultured for 24 h, 48 h, and 72 h 100 μL of MEM with 10% CCK-8 reagent was added to each well. The absorbance was recorded at 450 nm.

### 2.7 Scratch assay

L929 cells (5 × l0^5^ cells/well) were inoculated in the six-well plate. When L929 cells reached 100% confluence, the wound was created with a sterile 200 μL pipette tip. After the cells were washed with PBS and serum-starved for 5 h. The supernatant was replaced by the CS/APCS hydrogel leaching solution (0.075%v/v), or the AP solution (75 mg/L). After 0, 12, and 24 h, the scratched areas were photographed using an inverted microscope (Osaka, Japan) at ×40 magnification.

### 2.8 Experimental animals

32 male Sprague-Dawley rats (180–220 g) were obtained from Beijing HFK Bio-Technology Co., Ltd. Rats were housed under standard laboratory conditions (22°C ± 2°C, 55%–60% relative humidity, and 12 h light/12 h dark cycle) and the rats received food and water *ad libitum*. All the animal experiments were performed following the Ethics Committee of the Experimental Animal Research Centre, Shenyang Medical College (protocol No.SYYXY2023030501). After 1 week of adaptive feeding, rats were fasted for 12 h and then anesthetized by intra-peritoneal injection of 10% chloral hydrate to induce anesthesia. The dorsal surface hair of the animals was shaved, and a full-thickness skin defect with a diameter of 20 mm was created using scissors, then the rats were returned to their cages individually. Animals were randomly divided into four groups (n = 8 per group): the NC group received no treatment, 0.1 mL of CS for the group CS, 0.1 mL of AP for the group AP, and 0.1 mL of APCS for the group APCS, drugs were administered once during the entire treatment period. Photographs were taken with a digital camera on days 0, 4, 7, 10, and 14. In each group, four rats were randomly euthanized on days 7 and 14 after starting the treatment. The tissues collected from the healing area adjacent to the wound site and normal skin. The collected samples were cut into two parts, half of the samples at −80°C for further molecular analyses, and the others fixed in a 10% neutral formalin solution for histological analysis.

### 2.9 Histopathological assay

After paraffin embedding, the samples were cut into a 5 μm thick section and stained with hematoxylin and eosin for the study of tissue appearance. Masson’s Trichrome Staining Kit was used to detect the content of collagen in wound healing. Angiogenesis in wound healing was confirmed by immunohistochemistry with antibodies CD31 (1:2000) and α-SMA (1:5000). Briefly, each section was incubated in 3% H2O2 for 10 min and in anti-CD31 or anti-VEGF at 4°C overnight. And then the slice was incubated with Poly-HRP Anti Mouse/Rabbit IgG for 30 min at 37°C.

### 2.10 Western blot

Cells were seeded into 6-well plates at a density of 5 × 10^5^/well. After 24 h, the supernatant was replaced by the CS/APCS hydrogel leaching solution (0.075%v/v), or the AP solution (75 mg/L). Tissues or cells were homogenized in RIPA buffer, and the protein concentration of the supernatants was determined by BCA Kit. The samples were then moved to sodium dodecyl sulfate-polyacrylamide gel electrophoresis at 100 V and they were subsequently transferred to PVDF membranes at 80 V. Afterward, the membranes were blocked with skimmed milk for 1 h at room temperature. The membranes were with α-SMA (1:1000), VEGF (1:1000), PGP9.5 (1:5000), Catalase (1:1000), and GAPDH (1:50000) polyclonal primary antibodies at 4°C overnight. Corresponding anti-rabbit or anti-mouse secondary antibodies (1:3000) were incubated for 1 h at room temperature. The immunoreactive bands were developed with Clarity Western ECL Substrate and analyzed using ImageJ software.

### 2.11 Statistical analysis

All data were analyzed using GraphPad Prism 9 software (GraphPad, United States) and SPSS 23.0 software (IBM, United States). Results were expressed as mean ± SEM. Comparison between groups was conducted by one-way ANOVA, and P < 0.05 was considered significant.

## 3 Results

### 3.1 The characterization of APCS hydrogels

**FIGURE 1:**
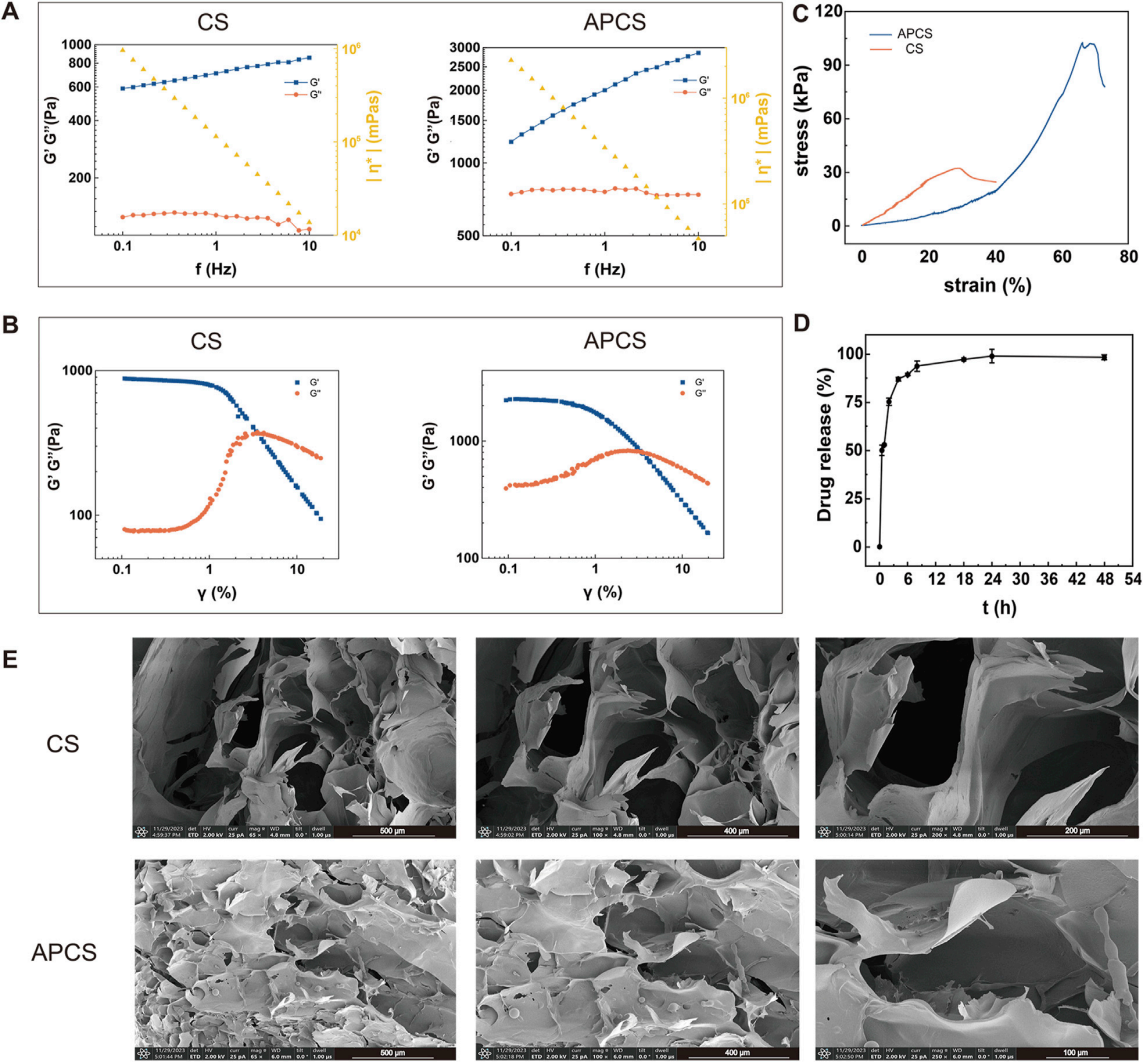
Performance characterization of APCS hydrogels. **(A)** Rheological measurements of CS and APCS hydrogels. **(B)** The change of the G′ and G″ of CS and APCS hydrogels with γ. **(C)** Stress-strain curve of different hydrogels. **(D)** Drug release measurements of APCS hydrogels. **(E)** Hydrogel microstructure imaged by SEM.

In order to investigate the behavior of the hydrogel, we examined the gelation kinetics of CS hydrogels and APCS hydrogels by analysis of the storage modulus (G′) and loss modulus (G″). The frequency sweep profiles demonstrated that the hydrogels exhibited a value of G′ of 600 Pa (CS) and 1200 Pa (APCS) at 0.1 Hz. And the values of G′ and G″ for APCS hydrogels were always higher than that for CS hydrogels at the same frequency. Moreover, as G′ exceeded G″ over the entire frequency range, CS hydrogels, and APCS hydrogels showed a gel state. These data suggest that the CS hydrogels loaded with AP could increase the elastic free energy (Figure 1A). Meanwhile, a strain sweep on CS hydrogel or APCS hydrogel was carried out from 0.1% to 10% at a frequency of 1 Hz to determine the linear viscoelastic range (LVER). The amplitude sweep test showed that APCS hydrogels exhibited a similar LVER, compared to pure CS hydrogels (Figure 1B). The hydrogels showed fluid characteristics (G″ >G′) with a strain of more than 5%. In the compression test of hydrogels, the anti-pressure ability of the CS hydrogels was significantly reduced in about 30% strain, while the APCS hydrogels could over 60%, the findings revealed that the compressive strength of the hydrogel was significantly enhanced following the composite AP to the CS hydrogels (Figure 1C). We detected the amount of drug released as a function of time by drug release experiment. After 12 h of sustained release, the cumulative drug release rate was over 90%, which showed that the APCS hydrogel could quickly release the medicines in a simulated wound condition. The drug release rate reached equilibrium after 24h, so the hydrogel had a good controlled release behavior for the drug (Figure 1D). The pore size of hydrogels is a critical parameter. A large porosity structure was observed in the CS hydrogel, while the APCS hydrogel showed a narrower range of porosity structure because of the crossing-linked of AP and the CS (Figure 1E). These results showed that the APCS hydrogel displays higher compressive strength than CS hydrogel and could improve the structural connections. Hydrogels’ structure with interconnected pore structures facilitates water absorption, oxygen and nutrient exchange, and cell migration and growth, providing conditions for the rapid healing of wound tissues.

### 3.2 APCS hydrogels promoted the proliferation of L929 cells

**FIGURE 2:**
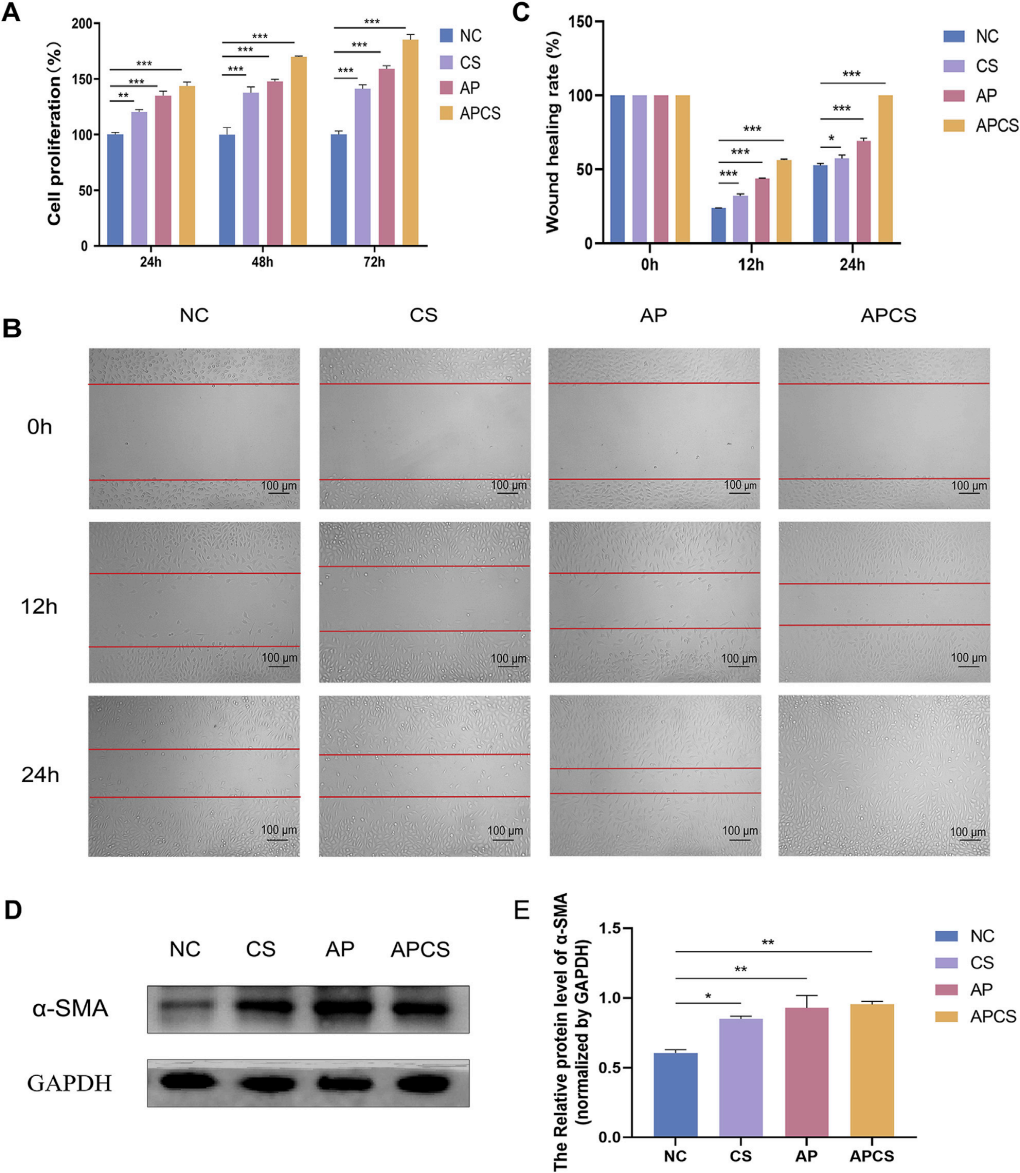
Cell proliferation, migration, and differentiation. **(A)** CCK-8 staining detected cell proliferation. n = 3. **(B)** The wound healing rate of cell migration areas (scale bar 100 μm). **(C)** The wound healing rate. n = 3. **(D)** Protein expression of α-SMA in fibroblasts. **(E)** Western blot analysis for α-SMA protein expression. n = 3. *P < 0.05, **P < 0.01, ***P < 0.001; error bars represent SEM.

To investigate the biocompatibility of APCS hydrogels, we detected the cell viability by CCK-8 staining. The results displayed that the cell viability was more than 95% in all concentrations, and there was no observed cytotoxicity in the APCS group at the maximum concentration. Then, the *in vitro* proliferation was evaluated on L929 cells. Compared with the NC group, the CS and AP groups could have different degrees of promoting fibroblast proliferation after treatment for 24 h, the proliferation rate was 120% ± 1.2% and 134.9% ± 2.4%, respectively. The proliferation rate of the APCS group (143.5% ± 2.1%) was significantly higher than the CS and AP groups. At 48 h post-treatment, the cell proliferation rate of the CS, AP, and APCS groups (137.5% ± 3.0%, 147.6% ± 1.1%, and 169.8% ± 0.4%) increased gradually. For cultivating 72 h, the fibroblast proliferation rate of the group treated with the APCS hydrogel was significantly higher than the NC and CS groups, and the data reached 185.3% ± 2.2% (Figure 2A). These results suggested that APCS hydrogels implied favorable biocompatibility, CS hydrogels and AP solution could have different degrees of promoting the proliferation of fibroblasts, and the APCS group displayed especially outstanding effects on increasing the proliferation of L929 cells.

### 3.3 APCS hydrogels promoted fibroblast migration

To assess the migration effects of APCS hydrogels on L929 cells, we examined the wound healing rate by scratch assay. After 12 h of CS hydrogels and AP solution treatment, different degrees of cell migration were observed at the edge of the scratch, the wound healing rate was 32.2% ± 1.9% and 43.8% ± 0.4%, respectively. The narrowest gap was observed in the APCS group (52.5% ± 0.8%). At 24 h post-treatment, the wound healing rates of the CS and AP groups were 57.5% ± 4.1% and 69.2% ± 3.3%, and the scratches were completely closed in the APCS group (100%). This indicated that CS hydrogels and AP solution could partly accelerate cell migration, and the APCS hydrogel was detected to significantly promote the migration of fibroblasts *in vitro* (Figures 2B, C). These data suggested that the application of hydrogel carriers helped AP play a better role in promoting the migration of fibroblasts.

### 3.4 APCS hydrogels induced fibroblast differentiation

The differentiation of fibroblasts into myofibroblasts plays an important role during the remodeling phase of wound repair, α-SMA is a crucial biomarker for fibroblast differentiation. In order to determine whether the APCS hydrogels induce the differentiation of L929 cells, we detected the production of α-SMA using Western blot analysis. The expression of α-SMA was rarely tested in the NC group, the level of α-SMA protein was elevated after treatment with the CS hydrogel, but it was significantly upregulated in the AP and APCS groups (Figures 2D, E). The results showed that treatment of AP solution and APCS hydrogels could effectively promote fibroblast differentiation *in vitro*.

### 3.5 APCS promoted healing of skin wounds

**FIGURE 3:**
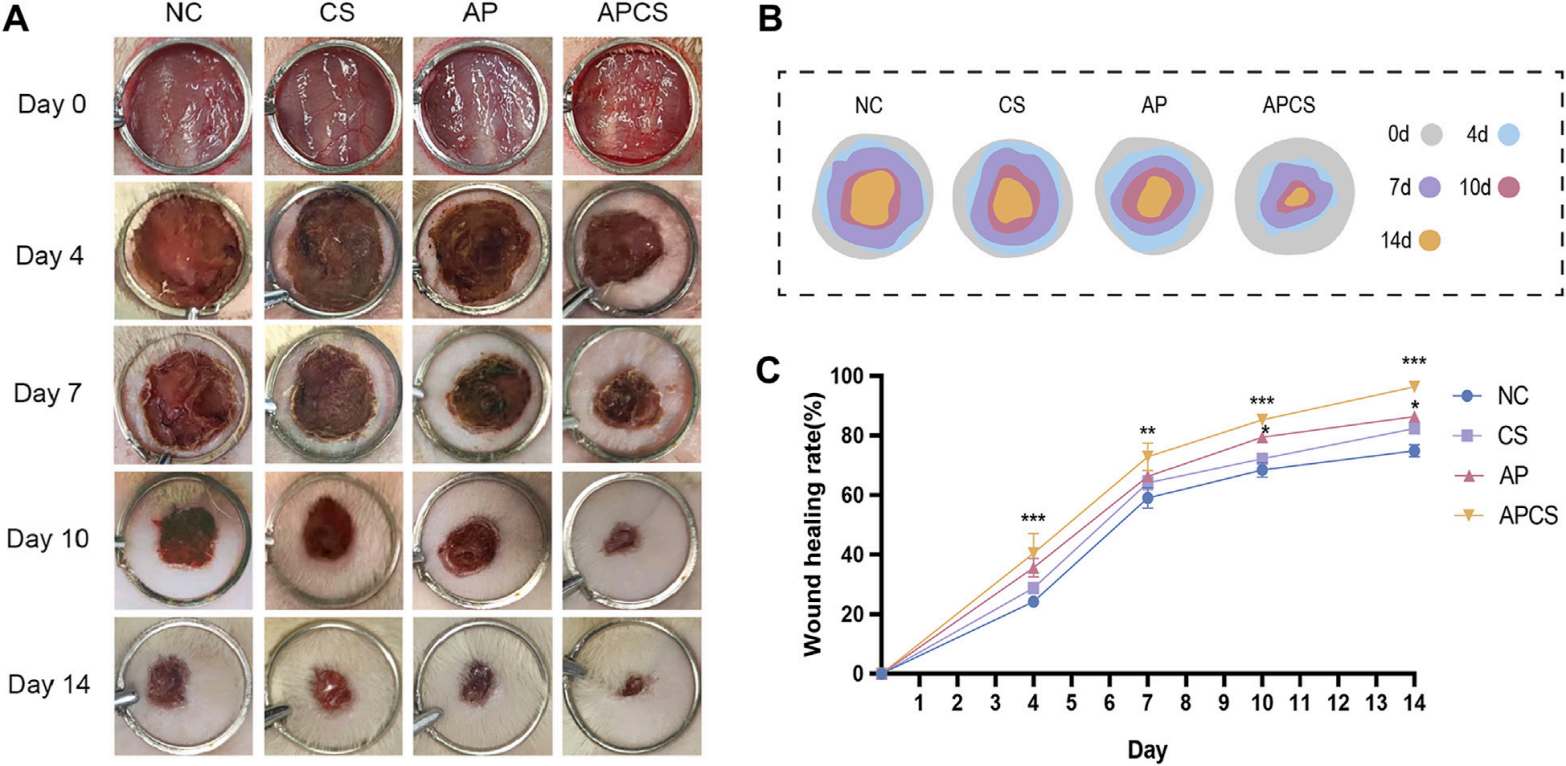
The effect of APCS on wound healing. **(A)** The pictures of wound healing. **(B)** The wound trace of four groups. **(C)** Wound healing rate. n = 4. *P < 0.05, **P < 0.01, ***P < 0.001; error bars represent SEM.

To investigate the therapeutic effects of the APCS hydrogel *in vivo* experiments, the full-thickness wounds were created on the backs of rats and treated with the CS hydrogel, the AP solution, and the APCS hydrogel, respectively. Photographs were taken on the 0, 4, 7, 10, and 14 days. During the entire treatment period, no significant differences in wound size were detected between the CS and NC groups. After the treatment of CS hydrogels for 10 days, the wounds of rats showed significant healing compared with the NC group. Notably, the APCS group cloud effectively accelerates wound closure during the entire healing process (Figures 3A–C). These results suggested that the application of the CS hydrogel carrier helped AP achieve better efficacy in promoting wound repair, the APCS hydrogel showed significantly promoting effects on wound healing.

### 3.6 Histological analysis of APCS hydrogels

**FIGURE 4:**
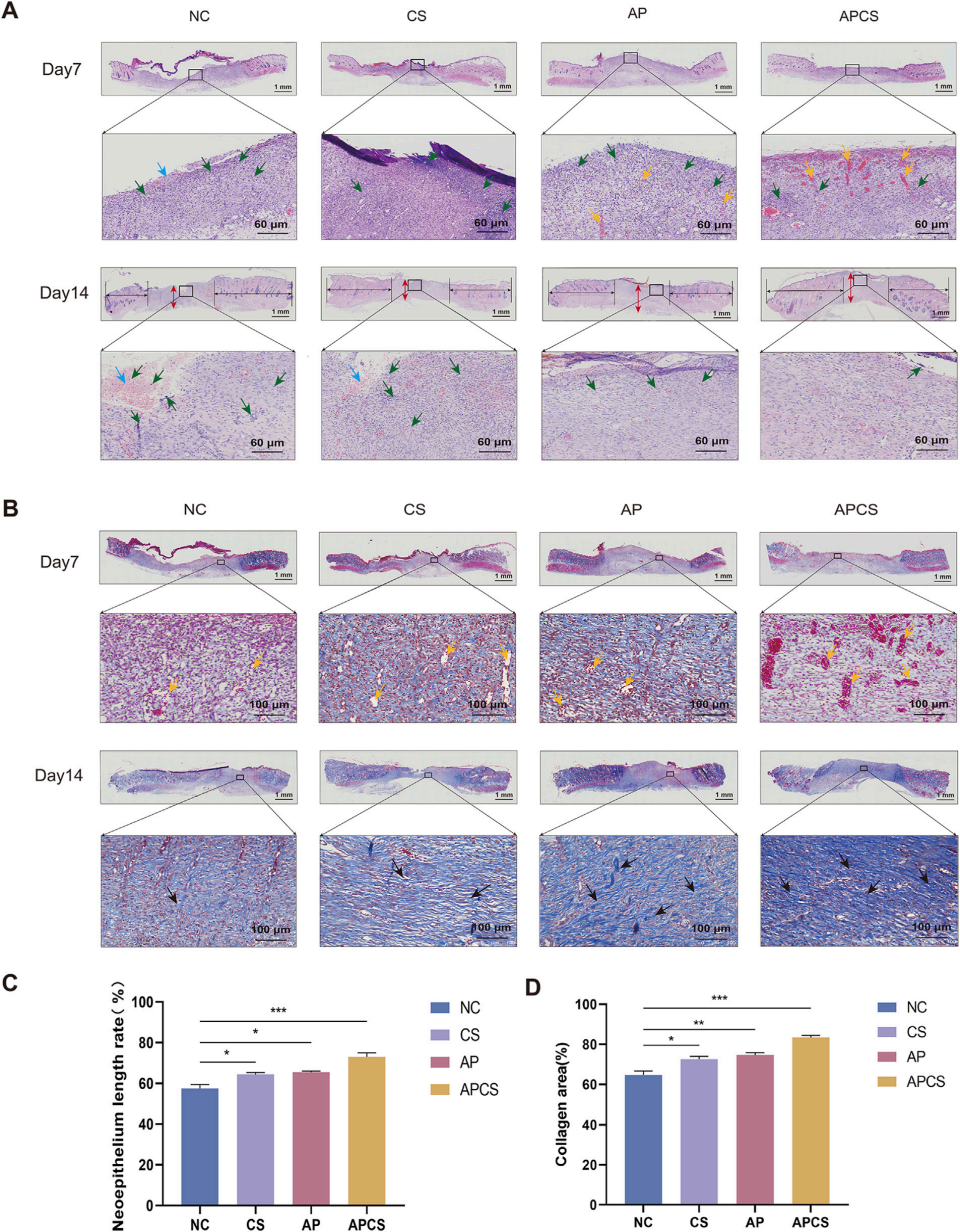
The effects of the APCS hydrogel on wound healing evaluated on the histological level. **(A)** Representative H&E-stained slides (scale bar 1000 μm), yellow arrow: blood vessels, red arrow: the thickness of the regenerated granulation tissue, green arrow: inflammatory cells, blue arrow: inflammatory exudate. **(B)** Masson’s trichrome staining images of the granulation tissue (scale bar 100 μm), yellow arrow: blood vessels, black arrow: collagen fiber. **(C)** The neoepithelium length rate of the wound. n = 3. **(D)** Collegen area of each group. n = 3. *P < 0.05, **P < 0.01, ***P < 0.001; error bars represent SEM.

Re-epithelialization and collagen synthesis have been considered important processes of wound healing. In the hematoxylin-eosin (HE) staining of wound tissue on the seventh day, different degrees of inflammatory reaction were observed in each group. Compared with the NC and CS groups, neovascularization was discovered in the AP group. In contrast to the AP solution treatment, more angiogenesis appeared in the APCS group. On the 14th day, the neoepithelium length in the CS and AP groups was significantly longer in comparison with that in the NC group, and the APCS group had a longer neoepithelium than the CS and AP groups. Furthermore, thicker granulation tissue was observed in the AP and APCS groups than in the NC and CS groups. As the wounds did not completely heal, many inflammatory cells and inflammatory exudate were found in the NC and CS groups. However, fewer inflammatory cells were discovered in the AP and APCS groups (Figures 4A, C). These results showed that AP solution and APCS hydrogels were able to facilitate angiogenesis, and the treatment of the APCS hydrogel shortened the transition time from the inflammatory phase to the proliferative phase and accelerated wound healing.

In the Masson staining of wound tissue on the seventh day, slight collagen deposition was observed in each group. After the treatment of CS hydrogels and AP solution, new blood vessels were formed compared with the NC group. Compared with the CS and AP groups, the extent of neovascularization was enhanced greatly in the APCS group. Trends in angiogenesis of each group were consistent with the changes observed in H&E staining on the seventh day. Compared with the NC group, more little and loose collagen bundles appeared in the CS and AP groups on the 14th day, and numbers of thicker and denser collagen fibers were observed in the APCS group (Figures 4B, D). These results displayed that the treatment of APCS hydrogels promotes collagen synthesis in the wound healing process.

### 3.7 APCS improved angiogenesis in wound

**FIGURE 5:**
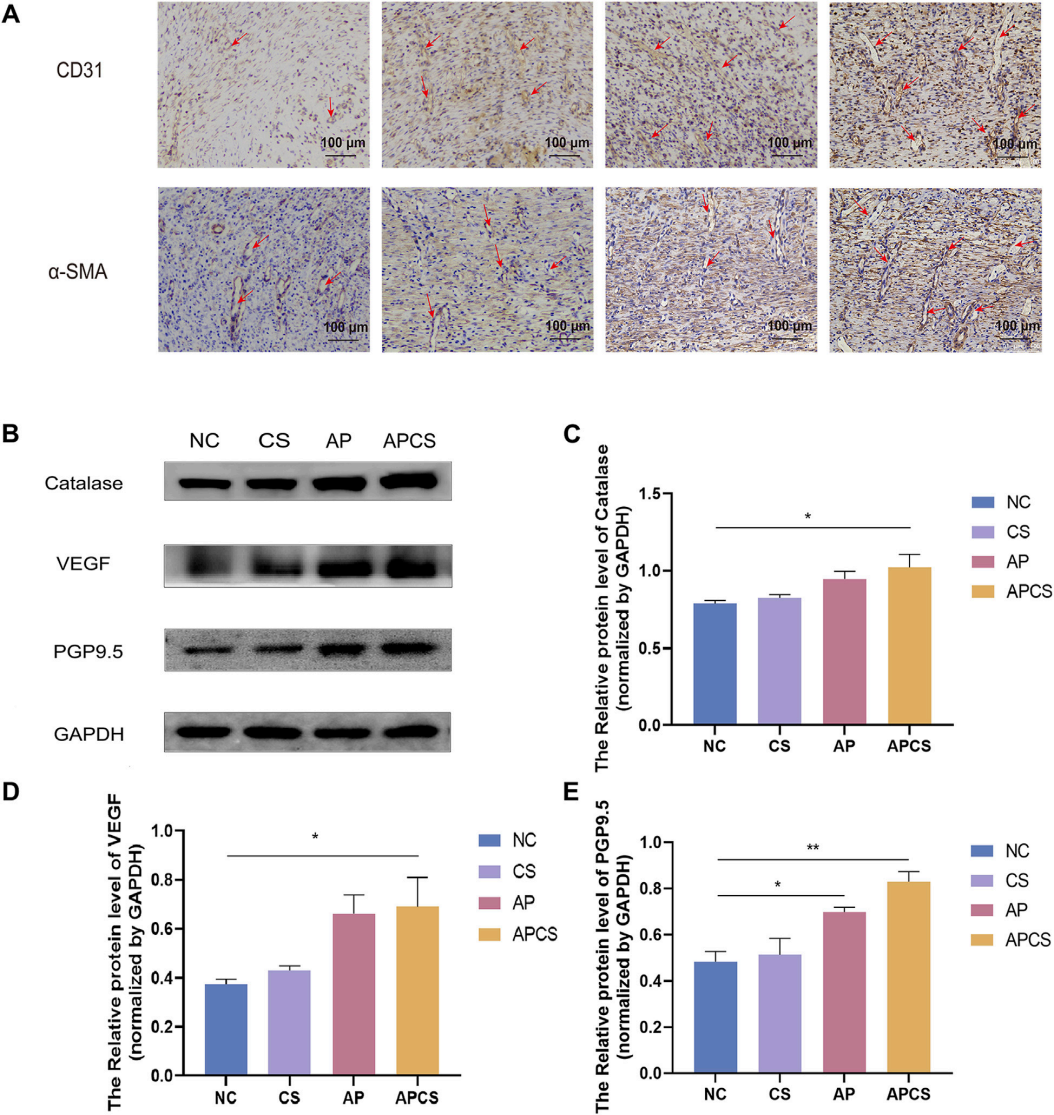
Mechanism research of wound healing. **(A)** Immunohistochemical staining of CD31 and α-SMA, the red arrow shows blood vessels (scale bar 100 μm). **(B)** Protein expression PGP9.5, VEGF, and Catalase in wound tissue. **(C)** Western blot analysis for Catalase protein expression. n = 3. **(D)** Western blot analysis for VEGF protein expression. n = 3. **(E)** Western blot analysis for PGP9.5 protein expression. n = 3. *P < 0.05, **P < 0.01, ***P < 0.001; error bars represent SEM.

CD31 is a marker of neovascularization in wound healing, and it is mainly expressed by vascular endothelial cells in early angiogenesis. In the immunohistochemical detection of wound tissue on the 14th day, a few CD31 were expressed in the NC group. The CS, AP, and APCS groups showed dense positive immunoreactivity, and the expression of CD31 in the AP and APCS groups was distinctly increased (Figure 5A). These results proved that the treatment of AP solution and APCS hydrogels promoted the regeneration of capillaries. α-SMA is recognized as an important biomarker of mature blood vessels in the proliferative phase of wound healing, and it is mainly expressed in the vascular smooth muscle cells during later angiogenesis. In the immunohistochemical detection of wound tissue on the 14th day, the expression of α-SMA was detected in each group, and a number of blood vessels were formed in the APCS group’s wound tissue compared with the CS and AP groups (Figure 5A). These results indicated that the APCS hydrogel could promote the regeneration of new blood vessels and mature blood vessels in the wound repair process.

### 3.8 APCS hydrogels promoted the expression of VEGF, PGP9.5, and catalase

High levels of ROS cause severe tissue damage in the wound healing process, and catalase eliminates excessive ROS in skin wound repair. We assessed the efficacy of the APCS hydrogel under oxidative stress and found no significant differences in catalase expression between the CS group and the NC group. Compared with the NC group, the expression of catalase was upregulated in the AP group, and the APCS group showed the highest level of catalase (Figures 5B, C). These results suggested that the treatment of APCS hydrogel could promote the elimination of ROS by upregulating the expressions of catalase. Moreover, angiogenesis plays an important role during the proliferative stage of wound healing, the increased expression of VEGF is necessary for the formation of new blood vessels during the tissue repair process. We found the VEGF level of the CS group was similar to the NC group by Western blot analysis. The expression of VEGF in the AP and APCS groups was significantly upregulated (Figures 5B, D). These results showed that the AP solution and the APCS hydrogel could accelerate the healing in rats by promoting angiogenesis. There is abundant nerve regeneration during the proliferative stage of wound healing. PGP9.5 is an important marker of epidermal nerves. After 14 days of treatment, no significant differences in the expression of PGP9.5 were discovered between the CS group and the NC group, and the expression level of PGP9.5 in the AP, and APCS groups was significantly increased (Figures 5B, E). These results displayed that the treatment of AP solution and APCS hydrogel could accelerate nerve regeneration by upregulating the expressions of PGP9.5.

## 4 Discussion

Wounds caused by physical and chemical injuries or microbial infection defects lead to long sessions and high expenditure, which places a heavy burden on physically and mentally for patients (Wang S. et al., 2023; Liu G. et al., 2024). Traditional Chinese medicine has shown wide experience in regulating the wound healing process and has displayed outstanding advantages in wound repair (Zhou et al., 2024; Chi et al., 2021; Yao et al., 2023). In traditional Chinese medicine, *A. membranaceus* and *P. notoginseng* are frequently used to promote skin wound healing (Jia et al., 2024; Gao et al., 2023). The outstanding carrier can help drugs sustainably release in wound healing and play a longer efficacy (Li et al., 2022). In this study, we encapsulated an extract of *A. membranaceus*-*P. notoginseng* in Carboxymethyl chitosan-Sodium alginate hydrogel and explored its efficacy on skin wound repair.

Hydrogels are natural or synthetic cross-linked polymers with a moisture content of more than 90% (Abdel-Mageed et al., 2022). CS hydrogel dressings provide a moist environment for cell migration and help the healing of the wound as their excellent water retention properties, adhesiveness, and injectability (Lv et al., 2019; Wang et al., 2021). The construction of hydrogel drug delivery systems is based on their porous network structure. The drugs in the hydrogel network avoid the influence of the external environment and effectively maintain the activity (Wathoni et al., 2024; Han et al., 2024). In previous studies, the hydrogel loaded with antimicrobial drugs and growth factors required for wound repair could appropriately control the release of drugs (Cui et al., 2020; Li et al., 2020). Our study demonstrates that APCS hydrogels with outstanding stability, biocompatibility, and the ability to release drugs rapidly could keep AP remaining on the wound longer and provide a beneficial healing environment.

In the early stages of wound healing, fibroblasts proliferate through mitosis, migrate into the wound bed under cytokine stimulation, and then transform into myofibroblasts to enhance the contraction ability of granulation tissue (Arif et al., 2021a; Talbott et al., 2022; Zhang et al., 2024). In previous studies, Astragalus polysaccharide and Panax notoginseng saponins promoted fibroblast proliferation in the wound healing process (Gao et al., 2023; Zhang et al., 2016). Our results suggested that the APCS hydrogel significantly promoted the proliferation and migration on L929 cell line. Myofibroblasts play an important role in the process of wound contraction (Monika et al., 2021). There are a large number of microfilament bundles present in the cytoplasm of myofibroblasts, which is a crucial skeleton structure of wound contraction. α-SMA abundantly expressed in myofibroblasts is involved in the formation of microfilament bundles and confers to myofibroblasts a high contraction ability (Arif et al., 2021b). Tight and extensive junctions are formed between myofibroblasts or cells and ECM through fiber complexes, gap junctions, and desmosomal junctions for promoting granulation tissue contraction and accelerating wound closure (Hinz et al., 2004; Schuster et al., 2023). Our data showed that AP solution and APCS hydrogels increase the expression of α-SMA, and induce fibroblast to myofibroblast differentiation.

*In vivo* studies of wound healing, the wound healing process of rats is slightly different from that of humans due to the presence of *panniculus carnosus* (Chen et al., 2015). However, the skin of rats is basically the same as that of human skin, which is divided into epidermis and dermis, and the wound of rats is easier to heal through re-epithelialization, which is similar to human wound healing. Therefore, the rat full-thickness wound model is often used *in vivo* studies of wound healing because it can better reflect the process of wound healing. In this study, the effect of APCS on wound healing was investigated by using the rat full-thickness wound model (Zomer and Trentin, 2018).

In the process of skin injury treatment, the efficacy of medicines is not fully exerted as they cannot remain on the wound for a long time (Mehnath et al., 2024). The outstanding carrier can help drugs sustainably release in wound healing and play a longer efficacy (Gao et al., 2023). Our study demonstrated that the treatment of AP solution had a limited therapeutic effect as it could not remain on the full-thickness wounds for a long time. However, the CS hydrogel loaded with the AP solution achieved a favorable effect during the entire treatment period in wound repair.

The repair of the wound is completed by contraction and re-epithelization of tissue (Cheng et al., 2021). In the early stage of wound healing, a large amount of granulation tissue is formed, and abundant collagen is secreted in fibroblasts from the granulation tissue. The wound area was filled with granulation tissue, collagen, and extracellular matrix (ECM) to create conditions for re-epithelialization (Luo et al., 2022a; Li et al., 2024). Previous studies showed that Panax notoginseng saponins, Astragalus polysaccharide, and CS hydrogels accelerated the formation of granulation tissue in wound healing (Yang et al., 2015; Wu et al., 2024; Li et al., 2023; Chen Y. et al., 2023). In our HE staining of wound tissue, the AP solution promoted wound healing by increasing the granulation tissue thickness, and the treatment of APCS hydrogel helped the AP solution play a better role in skin regenerative therapy. *Plenty of* collagen is secreted in fibroblasts from the granulation tissue after tissue damage (Taivanbat et al., 2023; Huang et al., 2022). Collagen fibers are involved in the wound healing process and help rebuild the damaged tissue through increased collagen fiber deposition (Pensalfini and Tepole, 2023; Kapoor et al., 2008). Our Masson-stained sections showed that the AP solution effectively promotes collagen synthesis. The application of CS hydrogel helped AP play a better role, and collagen bundles were arranged more regularly after the treatment of the APCS hydrogel. With the gradual wound healing, keratinocytes proliferate, migrate, and differentiate under the stimulation of various cytokines and growth factors (EGF, KGF, IGF-1, and NGF), to re-epithelialize in damaged skin and restore the epidermal barrier (Akita, 2019; Piipponen et al., 2020). In this study, the CS hydrogel and AP solution enhanced the re-epithelialization of wound tissue, and the combination of CS hydrogels and AP solution led to a better result in re-epithelialization.

In the full-thickness skin defects of wound tissue, vigorous cell metabolism produces a substantial amount of ROS, which is often accompanied by oxidative stress. Excessive generation of oxidative stress results in the aggravation of metabolic disorders in the wound area (Luo et al., 2021; Kant et al., 2014). Previous studies reported that *A. membranaceus* extract could promote the elimination of ROS by relieving tissue oxidative stress (Tam et al., 2014). In this study, the catalase expression level was not significantly increased after treatment with the AP solution, but the CS hydrogel loaded with the AP solution significantly upregulated the expressions of catalase and played an antioxidant role in the proliferative phase of wound healing. As the inflammatory phase finishes, the blood vessels and nerves are formed in wound tissue (Luo et al., 2022b; Li et al., 2021), and new capillaries in the granulation tissue provide oxygen and nutrients for cell metabolism (Smith and Rai, 2024). Our wound histology indicated that the APCS hydrogel could enhance angiogenesis and abundant blood vessels were formed in wound tissue. As a critical angiogenic growth factor, VEGF plays a vital role in tissue repair and regeneration (Liu Y. et al., 2024; Hu et al., 2021). Previous studies suggested that Astragaloside IV and Panax notoginseng saponins were able to facilitate angiogenesis, which was beneficial for wound healing (Luo et al., 2016; Lei et al., 2022). Our study found that the APCS hydrogel could significantly upregulate the expressions of VEGF compared with the AP solution alone. CD31 influences adhesion, contact, migration, and diapedesis of endothelial cells, and plays a crucial role in angiogenesis at early stages (Chen T. et al., 2023; Shukla et al., 2021; Kerstan et al., 2022). In the immunohistochemical detection of CD31, new blood vessels with clear lumen structures were observed in each group, among which the APCS group showed the highest level of neovascularization. Further confirmed that the treatment of APCS hydrogels could make AP solution play a better role in promoting vasculogenesis during the process of wound repair. With the gradual wound healing, capillaries convert into larger and more mature vessels. α-SMA is highly expressed by smooth muscle cells in vascular walls, which is regarded as the biomarker for the formation of mature blood vessels (Wang Y. et al., 2023; Yu et al., 2020). In our immunohistochemical detection of α-SMA, the APCS hydrogel played a more obvious role in promoting angiogenesis compared with the treatment of the CS hydrogel and AP solution alone. Our Western blot analysis and immunohistochemistry staining of wound tissue demonstrated that the expression of VEGF, CD31, and α-SMA significantly increased after the treatment of APCS, and the APCS hydrogel promoted the formation of new and mature blood vessels in the wound healing process.

The skin is a complex network with nerves, blood vessels, and specific receptors influenced by various physiological and disease processes (Ashrafi et al., 2016; Guo et al., 2023). The recovery of skin sensory function is an important indicator of cutaneous regeneration, various sensory nerve endings are widely distributed in the skin. Upregulated expression of PGP9.5 is a crucial feature in nerve endings regeneration (Van et al., 2016). A number of studies showed that astragaloside IV and panaxydol promoted nerve regeneration after sciatic nerve transection in rats (Zhang and Chen, 2013; Wang et al., 2022). Western blot analysis of the wound tissue showed that the treatment of AP solution promoted the expression of PGP9.5, and the application of hydrogel carriers helped AP play a better role in promoting nerve regeneration.

## 5 Conclusion

Overall, we fabricated a Carboxymethyl chitosan-Sodium alginate hydrogel loaded with *A. membranaceus*-*P. notoginseng* which could accelerate full-thickness wound healing with its favorable stability, biocompatibility, and the ability to rapidly release drugs. APCS hydrogels dramatically promoted fibroblast proliferation, migration, and differentiation into myofibroblast on L929 cell line. In a full-thickness SD rat wound model, APCS hydrogels reduced inflammatory response, facilitated the formation of granulation tissue and deposition of collagen fibers, and improved re-epithelialization of wound tissue. During the proliferative phase of wound healing, APCS hydrogels inhibited oxidative stress and promoted angiogenesis and neurogenesis. In conclusion, this APCS hydrogel possesses significant value for wound healing in rats and may be a potential therapy for treating full-thickness skin defect wounds.

## Data Availability

The raw data supporting the conclusions of this article will be made available by the authors, without undue reservation.
